# Low-Area Four-Channel Controlled Dielectric Breakdown System Design for Point-of-Care Applications

**DOI:** 10.3390/s22051895

**Published:** 2022-02-28

**Authors:** Jonggi Hong, Yeonji Oh, Hojong Choi, Jungsuk Kim

**Affiliations:** 1 Department of Health Sciences & Technology, Gachon Advanced Institute for Health Sciences & Technology, Gachon University, Incheon 21999, Korea; jonggi@bme.gachon.ac.kr; 2 Department of Medical Science, Korea University, Seoul 02841, Korea; ldypk3@korea.ac.kr; 3 Department of Eletronic Engineering, Gachon University, Seongnam 13306, Korea

**Keywords:** point-of-care, lab-on-a-chip, handheld device, molecular, multi-channel, low-area, transimpedance, nanopore, circuit design

## Abstract

In this study, we propose a low-area multi-channel controlled dielectric breakdown (CDB) system that simultaneously produces several nanopore sensors. Conventionally, solid-state nanopores are prepared by etching or drilling openings in a silicon nitride (SiNx) substrate, which is expensive and requires a long processing time. To address these challenges, a CDB technique was introduced and used to fabricate nanopore channels in SiNx membranes. However, the nanopore sensors produced by the CDB result in a severe pore-to-pore diameter variation as a result of different fabrication conditions and processing times. Accordingly, it is indispensable to simultaneously fabricate nanopore sensors in the same environment to reduce the deleterious effects of pore-to-pore variation. In this study, we propose a four-channel CDB system that comprises an amplifier that boosts the command voltage, a 1-to-4 multiplexer, a level shifter, a low-noise transimpedance amplifier and a data acquisition device. To prove our design concept, we used the CDB system to fabricate four nanopore sensors with diameters of <10 nm, and its in vitro performance was verified using λ-DNA samples.

## 1. Introduction

DNA sequencing technologies play an important role in analyzing DNA nucleotides and can be utilized in the medical and pharmaceutical fields to precisely diagnose diseases and provide personalized medicines [1,2]. Since the development of Sanger sequencing in 1977 [3], various sequencing methods have been devised to reduce processing costs and time. Recent DNA sequencing methods are based on polymerase chain reaction (PCR) techniques, which can amplify specific DNA targets into millions of copies. Individual nucleotides can be detected optically by combining them with fluorescent materials. For instance, solid-phase amplification is a representative DNA sequencer product that currently occupies more than 90% of the global genomic sequencing markets [4]. However, PCR-based sequencing methods can only support sequencing in short read lengths (100–150 bp) and require complex DNA sample preparation and processing procedures, all of which result in analyses that have high costs and low speeds. In addition, bulky sequencing devices will not be suitable for future point-of-care applications. Therefore, it is essential to develop time-, cost-, and area-effective sequencing methods.

**Figure 1:**
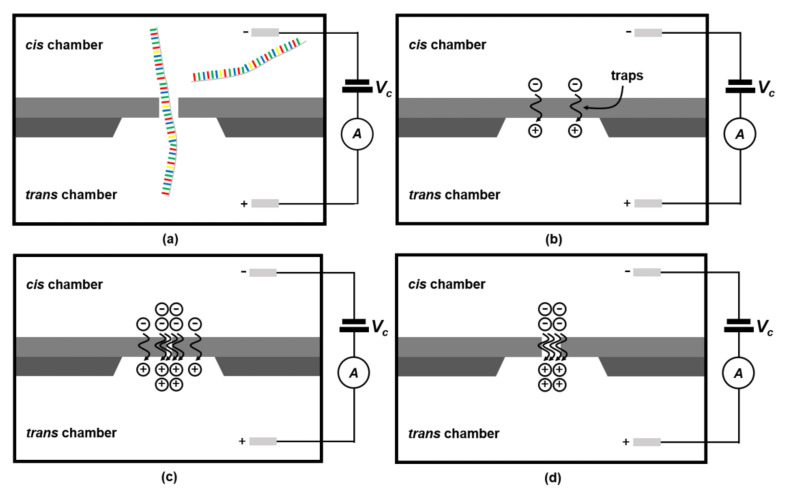
Nanopore formation using the controlled dielectric breakdown (CDB) technique. (**a**) Nanopore principle for DNA analysis; (**b**) leakage ionic current induced by an electric field resulting in a redox reaction and ionization; (**c**) dielectric breakdown occurring in an area with densely accumulated trapped electrons; (**d**) nanopore channel formation after CDB.

Nanopore sensors are among the most promising tools in next-generation DNA sequencing [5]. They are equipped with nanometer-sized holes that enable highly sensitive detection of biomolecules, such as nucleic acids and proteins, in a single molecular unit [4]. The small pore in each sensor allows DNA nucleotides to be analyzed with high precision, speed, low cost, and long read lengths [5,6]. Nanopore sensors (illustrated conceptually in Figure 1a contain the main chamber that is divided into *cis*- and *trans*-chambers, each filled with an ionic buffer. Upon applying a constant voltage (command voltage; *V_C_*) to the chambers, an ionic current will begin to flow across the nanopore channel, and the DNA sample, which has a negative charge at its backbone, will move from the *cis*-chamber towards the *trans*-chamber, via electrophoresis. When the DNA strand passes through the pore connecting the *cis*- and *trans*-chambers, the ionic currents can be modulated according to the DNA bases, which ultimately will induce different current magnitudes [7]. Finally, by detecting these differences, DNA nucleotides can be sequenced.

Nanopore sensors can be constructed using biological pores [8,9,10,11,12], which utilize naturally existing proteins, or by using solid-state nanopores [11,13], which are artificially created through the synthesis of nanomaterials. Biological pores can be formed by inserting a single α-hemolysin protein (α-HL), [8,10,12], which is secreted by *Staphylococcus aureus* and *Mycobacterium smegmatis* porin A (MspA) [9,10,12] into the lipid bilayer. Using α-HL nanopores, Oxford Nanopore Technologies has recently succeeded in sequencing DNA strands up to 4 M bp in length. These DNA sequencers have low costs, are by far the most portable DNA sequencers available, and can acquire data in real-time [14]. However, biological sensors are unstable and highly sensitive to experimental conditions, such as pH, temperature, and electrolyte concentration [12]. In addition, mass production of these sensors is difficult because they are handmade. In contrast, solid-state nanopores are constructed by harnessing several technologies, namely the focused ion beam (FIB), the focused electron beam (FEB), transmission electron microscopy-based drilling, and ion beam sculpting [15,16,17,18]. Currently, FIB [16] and FEB [17,18] are the most popular methods used for precisely fabricating nanometer-sized pores on solid substrates. However, these methods require long fabrication times [16], they have high costs, and they require large equipment, such as the Zeiss LEO 1540XB crossbeam workstation (Carl Zeiss Microscopy GmbH, Oberkochen, Germany) and the FRA − 2 × 1 − 1 electron gun (Kimball Physics Model) [17,18]. To overcome these challenges, the controlled dielectric breakdown (CDB) technique was introduced [19]. The CDB technique, which is cost- and time-effective, can be used to produce a small nanopore (i.e., ~2 nm at minimum) by applying a high electrical field to the solid substrate for a few seconds [20]. However, this CDB system only makes a single nanopore on the silicone membrane at a time, ultimately leading to different nanopore diameter sizes due the pore-to-pore variation resulting from repeated nanopore generation. This disadvantage would impede the acquisition of stable and constant DNA data from the nanopores. Therefore, it is necessary to produce multiple nanopore sensors, each with the same diameter, under the same experimental conditions. CDB systems have been introduced in previous studies [21,22], however, these system sizes are too large for point-of-care applications. In this study, we propose a low-area, four-channel automatic CDB system on a circuit board, which can fabricate four nanopores independently and simultaneously. In the proposed system, a low-noise amplifier, multiplexer (MUX), level shifter, and data acquisition board (DAQ) were optimally configured to control the nanopore diameter during fabrication. To prove the concept, the proposed system was designed and fabricated on a single printed circuit board (PCB) and demonstrated on a benchtop using silicon nitride (SiNx) solid-state membranes and λ-DNA samples.

## 2. Materials and Methods

### 2.1. CDB

The CDB technique forms a nanopore channel by supplying a constant potential difference (∆*V_C_*) to a SiNx membrane with nanoscale thickness. Thereafter, a high electric field induced by the potential difference generates continuous leakage of ionic current across the membrane. The leakage current (*I_N_*) results in a redox reaction and ionization on the surface, producing free electrons (see Figure 1b) [19]. The number of trapped electrons on the SiNx membrane (see Figure 1c) is proportional to the magnitude of *I_N_*. Finally, the dielectric breakdown phenomenon occurs in the areas where trapped electrons are densely accumulated, resulting in the formation of nanopores (see Figure 1d).

Typically, <10 nm solid-state nanopores are used for DNA analysis. This is owing to the fact that the pore diameters are inversely proportional to the signal-to-noise ratios of the ionic current signature captured by the nanopore channels (see Figure 1a) [23]. As such, recent research on CDB nanopore formation has focused on achieving diameters of <10 nm [24]. Additionally, implementing high-throughput data acquisition for accurate DNA analysis is essential. As such, CDB systems should produce multiple nanopore sensors of equal diameter to minimize the extensive pore-to-pore variation that arises from different experimental conditions. However, to the best of our knowledge, a multiple-channel automatic CDB system is yet to be published. Therefore, in this present study, we aimed to implement a four-channel CDB system that can independently produce pores with diameters of <10 nm. Finally, we aimed to verify these nanopore sensors using λ-DNA samples (D-2510; BIONEER Inc., Daejeon, Korea).

### 2.2. Proposed Four-Channel CDB System

**Figure 2:**
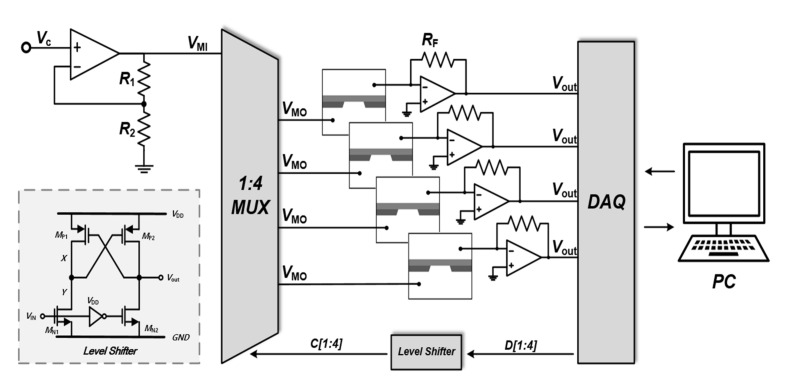
Schematic diagram of the proposed four-channel CDB circuit.

The proposed four-channel CDB system architecture comprises low-noise transimpedance amplifiers that measure the ionic *I_N_* from the SiNx membrane, a non-inverting amplifier that boosts the *V_C_* for high electric field generation, a level shifter that provides a voltage-level interface between the DAQ and the MUX, and a 1-to-4 MUX to independently cuts off *V_C_* applied to the nanopore sensors (Figure 2). Finally, the DAQ plays a controlling role in generating *V_C_*, by selecting the MUX channel, digitizing the amplified leakage signal, and providing the data to a computer to be used in estimating and adjusting the nanopore sizes.

It is important to note that in forming a nanopore on the SiNx membrane, the DAQ needs to generate *V_C_* with a wide range (i.e., 0–20 V). The wide voltage range is required because of the different membrane materials and thicknesses. More specifically, thicker membranes require stronger electric fields. However, the DAQ can only provide a maximum output voltage of 10 V, which is insufficient for nanopore formation on thick membranes. As such, to increase the *V_C_* range to 20 V, a non-inverting amplifier (AD820, Analog Devices, San Jose, CA, USA) with a voltage gain of 1 + *R*_1_/*R*_2_ was employed in this CDB design, where the resistance values for *R*_1_ and *R*_2_ were each set to 10 kΩ. As a result, the amplifier output voltage could meet the required a range of 0–20 V. The increased *V_C_* (hereafter, *V_MI_*) was then applied to a 1-to-4 MUX (Multiplexer, CD4066B, Texas Instruments, Dallas, TX, USA).

The MUX cuts off the +MUX output voltage (*V_MO_*) applied to each *trans*-chamber. When one of the SiNx membranes displays larger pores than the other membranes, then the *I_N_* in that specific membrane (see Figure 1) will also be higher than that of the other membranes. Accordingly, the transimpedance amplifier (TIA) output determined by *I_N_* × *R_F_* will also exceed the preset threshold. At that moment, the DAQ will generate control signals (*D*_1_, *D*_2_, *D*_3_, and *D*_4_) to turn off the switch in the 1-to-4 MUX. Here, the digital control signals fed into the MUX should have high pulse magnitudes, similar to the *V_MO_* variation of up to 20 V, as this allows its switches that comprise high-voltage transistors to be fully turned on. However, the DAQ can only provide an output of 5.03 V, which is insufficient for MUX control. Thus, to address this voltage difference, we configured a level shifter (Figure 2) to amplify the DAQ digital output waveforms of 5.03 to 20 V, without any phase shift. Here, the transient waveforms for *D*_1_, *D*_2_, *D*_3_, and *D*_4_ are determined by a finite-state machine (FSM) that is realized using the LABVIEW program Version NXG 5.1. A detailed description of the FSM will follow shortly.

*V_MO_* is applied to the *trans*-chambers of the SiNx membranes, while the *cis*-chambers are connected to the inverting input of the TIAs. According to the electric field equation: *E* = ∆*V*/*T*, where ∆*V* and *T* indicate the voltage the difference between the *cis*- and *trans*-chambers and the thickness of the SiNx membrane, respectively. A high electric field is formed across the SiNx membrane, resulting in a dielectric breakdown (illustrated in Figure 1). As soon as the nanopore channel is generated, *I_N_* abruptly increases from a femto-ampere current to a few hundred picoamperes. This abrupt increase is continuously monitored by the DAQ. Accordingly, *I_N_* must be converted from a picoampere range to a readable voltage range because the DAQ analog input with 16-bit resolution can only sense voltage. Therefore, a TIA with adjustable feedback resistors (i.e., 1, 2, 4, and 12 MΩ) was used to convert and amplify the minute current to a readable voltage range.

The TIA should achieve low input noise to precisely sense *I_N_* variation from the nanopore sensor. As such, the resistive-feedback TIA designed in this study has input-referred current noise defined as follows [25]:

(1) $$S_{H}\left(f\right)\approx {\left[\frac{1}{R_{F}}+\frac{1}{R_{N}}+2\pi f\cdot C_{N}\right]}^{2}\underline{V_{n,AMP1}^{2}\left(f\right)}+\frac{4KT}{R_{F}}$$

where *R_F_*, *R_N_*, and *C_N_* denote the feedback resistor of the TIA, the mean nanopore resistance, and the capacitor, respectively, and *V*^2^*_n_*_,*AMP*1_ represents the core amplifier (AD549, Analog Devices, San Jose, CA, USA) input noise voltage with 35 nV/Hz. According to Equation (1), the resistive-feedback TIA input-referred current noise can be decreased by increasing the feedback resistance. However, the stray capacitance of the high feedback resistance causes a phase delay. Therefore, it is important to select the appropriate feedback resistance. According to our heuristic experiment, when the maximum input voltage was applied, an ionic current of 0.78 μA flowed in the nanopore with a size of approximately 10 nm. Therefore, the maximum value of the feedback resistor was set to 12.82 MΩ to satisfy the DAQ analog input range of 10 V. With respect to the 16-bit resolution of the DAQ input analog-to-digital converter, a feedback resistance of 2 MΩ was set. In addition, 5th-order Bessel filters with a 10 kHz cut-off frequency were used to further reduce the high-frequency input current noise. Accordingly, the resistive-feedback TIA had an input-referred current noise of 9.3228 pA_RMS_ in a bandwidth of 10 kHz. In this work, the low-noise TIA is used to only measure the leakage current generated from the SiNx membrane. By adding a difference amplifier after the TIA, we will be able to configure a high-gain DNA readout system in the future.

To automatically control the nanopore diameter, we built an FSM using the LABVIEW software Version NXG 5.1. The main function of the FSM was to monitor *I_N_* change and send a flag signal comprised of *D*_1_, *D*_2_, *D*_3_, and *D*_4_ to the MUX through the DAQ, when the nanopore diameter was larger than the predetermined size. The nanopore diameter formula (*D_PORE_*) was determined as follows [26]:

(2) $$G=\frac{1}{R_{N}}=\frac{I_{N}}{V_{MO}}=\sigma [\frac{4L_{PORE}}{\pi D_{PORE}^{2}}+\frac{1}{D_{PORE}}{]}^{-1}$$

(3) $$D_{PORE}=\frac{I_{N}+\sqrt{I_{N}\left(\frac{16\sigma L_{PORE}V_{MO}}{\pi }+I_{N}\right)}}{2\sigma V_{MO}}=2\sqrt{\frac{I_{N}L_{PORE}}{\sigma \pi V_{MO}}}+\frac{\;I_{N}}{\sigma V_{MO}}$$

where *G* is the nanopore conductance, *V_MO_* is the MUX output voltage applied to the SiNx membrane, *L_PORE_* is the length of the cylindrical nanopore, and $\sigma \left(10.5\;{\mathrm{Sm}}^{-1}\;\mathrm{at}\;23\;{}^{\circ}C\;\right)$ [27] is the surface charge density in the membrane. In Equation (3), only two parameters, namely *V_MO_* and *I_N_* are subject to change, whereas the other parameters are dependent on the characteristics of the specific SiNx membrane. Ultimately, this means that the nanopore diameter is adjustable by monitoring the *I_N_* change and controlling *V_C_* in relation to *V_MO_*.

**Figure 3:**
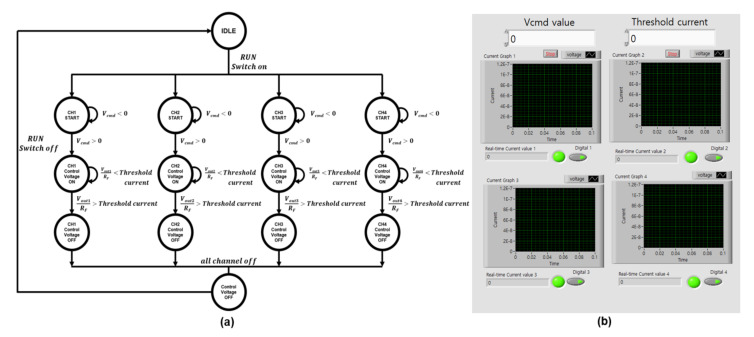
(**a**) A finite-state machine (FSM) algorithm adopted to monitor ionic leakage current (*I_N_*); (**b**) user interface window customized using the LABVIEW software Version NXG 5.1.

An FSM algorithm was employed to automatically control the nanopore size (see Figure 3a). Initially, when all the MUX switches are fully turned on, *V_C_* is slowly increased, and *I_N_* is observed from all SiNx membranes. When *I_N_* reaches the preset threshold, a flag signal is transferred to the MUX through the DAQ and the MUX switch is turned off, cutting off the supply of *V_MO_* and halting nanopore growth. Ultimately, the FSM designed here helps reduce the pore-to-pore variation that arises during the production of multiple nanopore sensors. Finally, we customized a LABVIEW program Version NXG 5.1 to monitor *I_N_* and control *V_C_* in real time (see Figure 3b).

## 3. Results and Discussion

### 3.1. Experimental Setup

U1 was the non-inverting amplifier utilized to generate *V_MI_* (see Figure 2). U2, U3, U4, and U5 denote the resistive-feedback TIAs that comprised the core amplifier of the AD549. These TIAs monitored the picoampere *I_N_* and sent an amplified voltage to the DAQ. U10 was the MUX that received the amplified *V_C_* (see *V_MI_* in Figure 2) and applied it to the four *trans*-chambers in the SiNx membrane. The MUX was controlled by an FSM through the DAQ. U11 was the level shifter that was used to increase the DAQ digital output from 5.03 V to 20 V. Because of this, the MUX switches could be fully turned on. U13, U14, U15, and U16 were 5th-order Bessel filters used to reject high-frequency input current noise.

**Figure 4:**
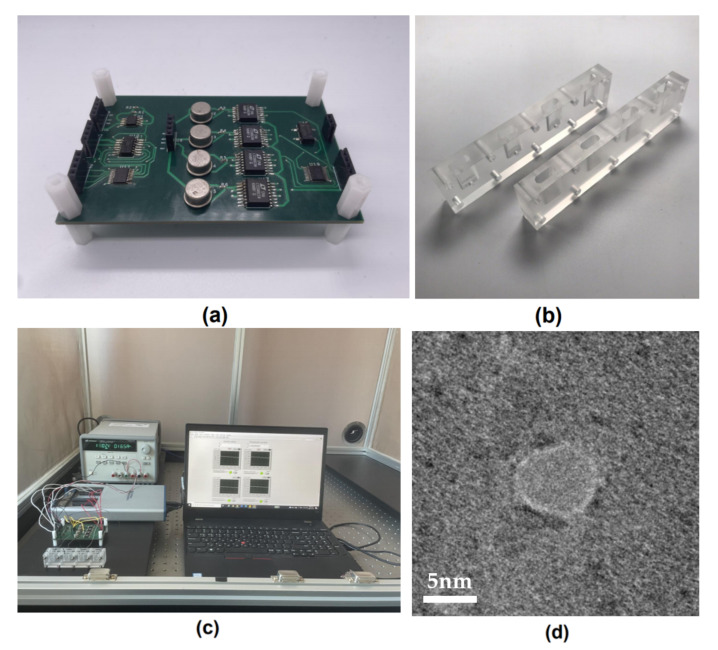
(**a**) Photograph of the proposed CDB system, with an area of 57 cm^2^; (**b**) customized flow cells with four *cis*-chambers and one *trans*-chamber; (**c**) complete CDB experimental setup; (**d**) nanopore microphotographed via TEM (Transmission Electron Microscopy).

The *trans*-sides of the customized four-channel flow cell were connected to the MUX outputs, whereas the *cis*-sides were linked to the TIA inverting inputs (Figure 4b). In addition, a SiNx membrane was inserted at the center of the flow cell. For this experiment, we utilized 12 nm thick SiNx membranes produced by NORCADA (Alberta, AB, Canada). To prevent 60 Hz ground noise interference, we placed all devices, including the CDB system, flow cell, DAQ, power supply, and laptop inside a Faraday cage, and employed a universal power supplier to further prevent ground noise interference.

### 3.2. Experimental Results

**Figure 5:**
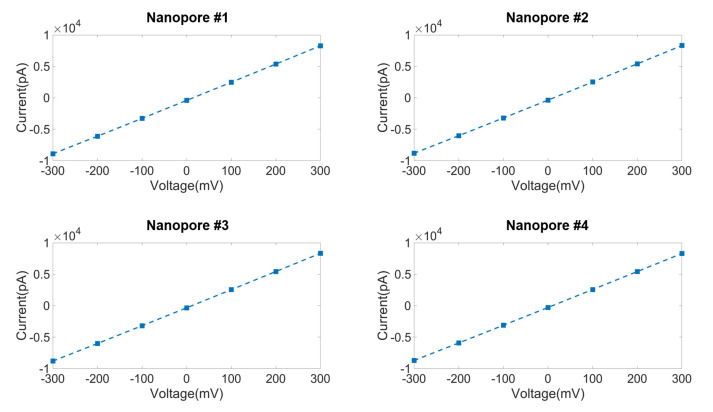
Current-voltage characteristic (IV) curves of four nanopore sensors fabricated by the CDB system designed in this study (Nanopore 1, 2, 3, and 4 have 28.65, 28.53, 28.55, and 28.39 nS, respectively).

Using the previously described four-channel CDB system with SiNx membranes, we fabricated four nanopore sensors with diameters of <10 nm, to be used for DNA analysis. *V_C_*, which varied from 7 to 10 V according to the pore formation size, was applied to the *trans*-chambers for a few seconds. After all the MUX switches were turned off, we removed the CDB system and measured the current-voltage characteristic (IV) curves of each nanopore, using the gold standard for nanopore experiments, namely the Axopatch 200 B amplifier (Molecular Device, San Jose, CA, USA). The results shown in Figure 5 indicate that nanopores 1, 2, 3, and 4 displayed conductances of 28.65 nS (34.90 MΩ), 28.53 nS (35.05 MΩ), 28.55 nS (35.02 MΩ), and 28.39 nS (35.22 MΩ), respectively. We estimated the nanopore diameters by substituting these conductance values into Equation (3). These results indicate that nanopores 1, 2, 3, and 4 had diameters of 7.74, 7.72, 7.72, and 7.70 nm, respectively (mean, 7.72 nm; standard deviation, 1.6%), which are satisfied with sub-10 nm nanopore goal required for DNA analysis. When the command voltage is applied for longer than 10 s, the pore size was maximized to ~130 nm in this experiment.

Figure 4d illustrates a microphotograph of the nanopore produced in the SiNx membranes using the proposed CDB system. Although four nanopore sensors were fabricated in this experiment, only one nanopore channel could be captured as a microphotograph. This was owing to the difficulty in determining the nanopore channel locations in the SiNx membrane.

**Figure 6:**
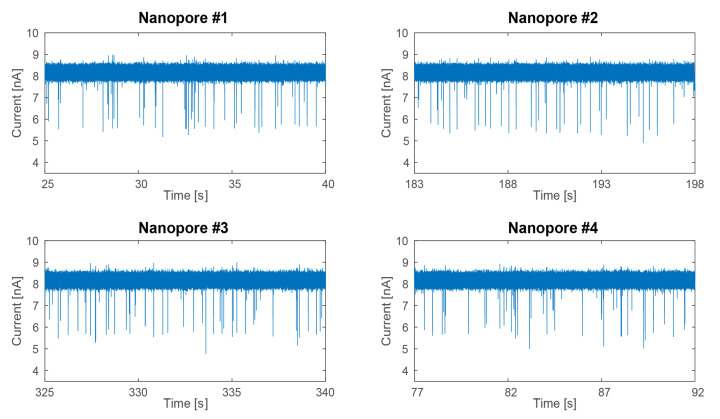
Observed DNA translocations with an average current variation of 1.6 nA.

To verify the performance of the nanopore sensors fabricated by the four-channel CDB system, we conducted a DNA translocation experiment. For the in vitro test, we used a buffer (pH 8.0) comprising 1 M KCl, 10 mM Tris-HCl, and λ-DNA (D-2510; BIONEER Inc., Daejeon, Korea). The measured transient results were acquired with a *V_C_* of 300 mV being applied to the nanopore sensors (Figure 6). Due to the four nanopore sensors having a similar pore diameter of approximately 7.70 nm, the open-channel currents were all set at 8.5 nA. The average translocation current variations for the four nanopore sensors were 1.6, 1.6, 1.7, and 1.6 nA, respectively. These results indicate that the four-channel CDB system designed here can accurately fabricate <10 nm nanopores to be used for various nanopore applications. We anticipate that the methodology outlined here will soon replace several methodologies that are currently being used to fabricate nanopore channels, namely the FIB, FEB, transmission electron microscopy-based drilling, and ion beam sculpting methods. The proposed CDB system in this work has only a function to fabricate a nanopore. In the future, we will refine the current CDB system by adding a readout function in order to accurately monitor DNA molecules.

## 4. Conclusions

In this present study, we proposed and designed a low-area, four-channel on-board CDB system to be used for various nanopore applications and demonstrated its ability to produce four nanopore sensors with an average diameter of approximately 7.70 nm. To reduce the pore-to-pore diameter variation that arises from conventional nanopore fabrication, we used four low-noise TIAs to monitor real time *I_N_* changes in the SiNx membranes, a 1-to-4 multiplexer to adjust the nanoscale aperture diameter, and a level shifter to provide an interface between the data acquisition device and the multiplexer. In addition, the LABVIEW software Version NXG 5.1 was employed to develop an FSM algorithm for a predetermined threshold and to program a customized user interface for TIA output monitoring. The CDB system proposed here occupied a board area of 57 cm^2^ and consumed 5.52 W of power. By applying a *V_C_* of 7–10 V across four SiNx membranes, we fabricated nanopore channels that were used to detect DNA translocation signals using an open-channel current of 8.5 nA and a blockade current of 1.6 nA. This in vitro test result indicates that the proposed CDB system can fabricate multiple nanopore sensors with low cost, high speed, and high throughput. To realize a hand-held DNA analyzer, we plan to reduce the active area and power dissipation of the system and increase the number of channels over two-fold, by integrating all the CDB systems into a single silicon chip.

## Data Availability

The data presented in this study are included in this article.
